# Nutritional rehabilitation strategies in abdominal surgery

**DOI:** 10.1097/MCO.0000000000001216

**Published:** 2026-02-18

**Authors:** Sawsan Abdul-Hamid, Wayne Fradley, Bethan E. Phillips

**Affiliations:** a Centre of Metabolism, Ageing and Physiology, School of Medicine, University of Nottingham; b Department of Surgery, Royal Derby Hospital, Derby, UK

**Keywords:** abdominal surgery, immunonutrition, malnutrition, rehabilitation, surgical outcomes

## Abstract

**Purpose of review:**

Major abdominal surgery imposes significant physiological and psychological stress on patients and can lead to associated changes with consequent morbidity. Postoperative rehabilitation aims to minimise the impacts of surgical stress and facilitate convalescence and return to normal function. This review aims to highlight recent research in abdominal surgery rehabilitation with a focus on nutrition and adjuvant exercise and best practice guidelines.

**Recent findings:**

Emerging interventions for restoration of normal gastrointestinal function postoperatively include the use of transcutaneous electrical acupoint stimulation and probiotics. Oral nutritional supplements in the postoperative period mitigate body weight loss, and routine use is now recommended after colorectal operations. Where oral intake is insufficient, enteral or parenteral nutrition is indicated, with meta-analyses demonstrating potential benefit of early supplementary parenteral nutrition. Immunonutrition postoperatively has been shown to reduce surgical complications and to have benefits over and above standard nutritional therapy. The evidence for adjuvant contractile interventions in the postoperative period is marked by heterogeneity in patient populations and exercise type, and further evidence is required in this space.

**Summary:**

The evidence base for postoperative rehabilitation is continually increasing, and recent international guidelines recommend several novel nutritional practices, notably the use of probiotics and immunonutrition.

## INTRODUCTION

Surgery *per se* imposes substantial physiological and psychological stress on patients, contributing to potential morbidity and mortality. In addition, gastrointestinal resections can lead to profound anatomical and physiological alterations, which can have detrimental effects on the nutritional status of patients both in the short and longer-term [1]. This risk is amplified given that those presenting for such operations are often at high risk of malnutrition even before surgery [2,3]. The postoperative period is frequently marked by inadequate dietary protein and energy intake [4] as well as immobility [5]. This combination results in rapid body weight and skeletal muscle (SKM) losses that may not completely recover even months after surgery [6]. Considering the advanced age of many of these patients, it needs to be recognised that malnutrition and sarcopenia are distinct but clearly related phenomena, each associated with poor surgical outcomes in a variety of settings [2,3,7,8]. Indeed, postoperative SKM losses have been shown to be associated with reduced overall and disease-free survival after cancer resection [9,10]. Thus, mitigation of inadequate nutritional intake and muscle preservation are amongst key goals of rehabilitation interventions.

Innovations in surgical techniques, such as minimally invasive approaches, have played a crucial role in attenuating surgical stress. Nonetheless, the postoperative period is still a key opportunity to optimise recovery profiles. Work by Danish surgeon Henrik Kehlet [11] introduced the concept of ‘Enhanced Recovery After Surgery’ (ERAS) in the late 1990s, with subsequent formation of the ERAS Society facilitating the systematic implementation of comprehensive guidelines underpinned by a substantial body of evidence. ERAS guidelines have thus narrowed the gap between traditional surgical care and evidence-based best practice. They provide standards of surgical, anaesthetic and multidisciplinary care in the peri-operative period to facilitate convalescence and minimise postoperative morbidity. Based on the concept of ERAS, important international surgical guidelines released in the last two years include the 2025 colorectal ERAS guidelines [12^▪▪^] and the European Society for Clinical Nutrition and Metabolism (ESPEN) surgical guidelines [13^▪▪^], which are highlighted in the discussion section below. 

**Box 1:**
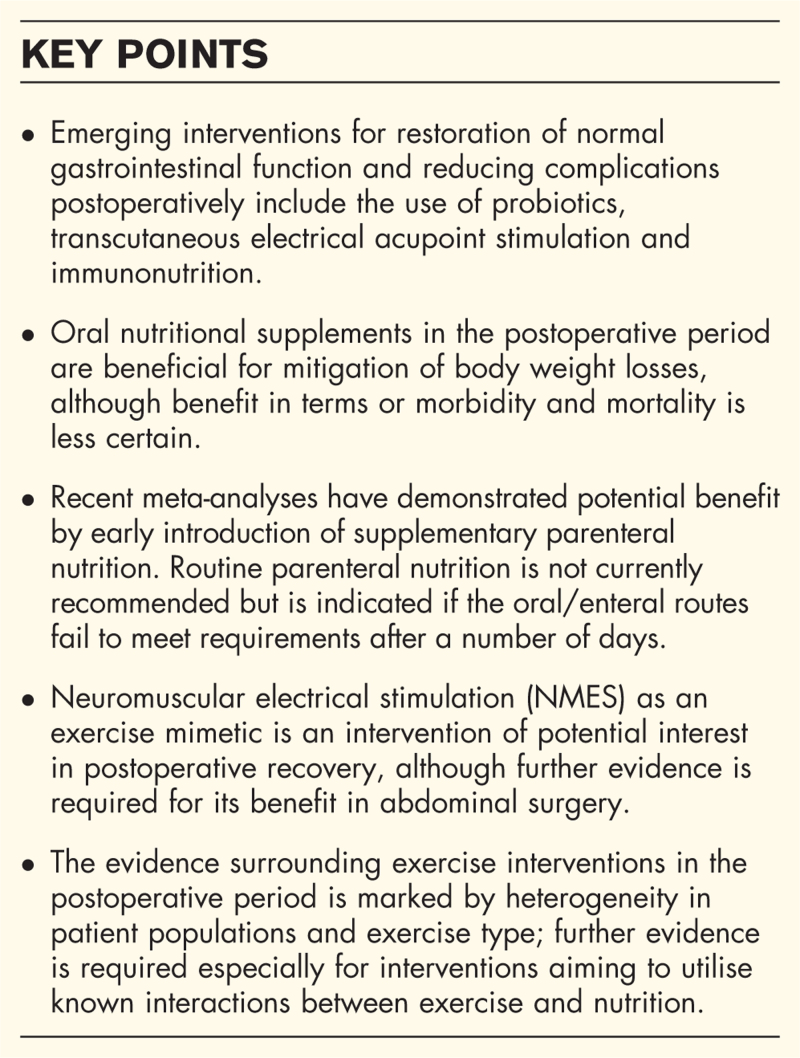
no caption available

## NUTRITIONAL STRATEGIES FOR EARLY RESTORATION OF GASTROINTESTINAL MOTILITY

Paralytic ileus refers to a phenomenon of delayed bowel motility that can cause delays in re-establishment of nutritional intake early in the postoperative course after major abdominal surgery. Whilst it is typically short-lived, it can be prolonged especially in the presence of surgical complications. Strategies to prevent or shorten this and re-establish gastrointestinal function are amongst key goals of early postoperative care. In this regard, early introduction of oral feeding has historically been a key component of ERAS guidelines. Indeed, the most recent (2025) ERAS guidelines for elective colorectal surgery advocate oral nutritional intake within 24 h after surgery [12^▪▪^]. In support of this, a recent meta-analysis exploring the impact of early oral feeding in colorectal surgery has confirmed its association with reduced rates of anastomotic leak, decreased time to flatus and reduced overall complications [14]. However, a higher rate of vomiting and need for nasogastric tube re-insertion was noted, leading the authors to recommend cautious consideration of the findings and potential need for refinement of future ERAS protocols. A cohort study looking at early oral feeding postgastrectomy in younger versus older patients has found no significant differences between the two groups in tolerability [15]. Early oral feeding after oesophagectomy is more controversial. Whilst recent systematic reviews have demonstrated no increased rates of anastomotic leak with early oral feeding, poorer nutritional outcomes were seen with early oral feeding compared to a combination of late oral and early enteral nutrition (EN) [16,17]. Current ERAS guidelines still advocate for early establishment of enteral (postanastomotic) feeding in this context [18].

Whilst intake of coffee and chewing gum are commonly used postoperatively to encourage gut motility, only recently have these low-risk interventions made their way into official ERAS guidelines [12^▪▪^]. These recommendations are supported by a recent network meta-analysis showing evidence for reduced time to defecation and reduced length of hospital stay (LoS) for both coffee and chewing gum [19]. The 2025 colorectal ERAS guidelines include further recommendations for a number of established adjunct measures for the prevention of ileus such as optimal analgesic approaches, the use of laxatives and prokinetics, and more novel interventions such as transcutaneous electrical acupoint stimulation (TEAS) [12^▪▪^]. TEAS entails the use of surface electrodes to stimulate acupoints by applying electrical currents via the skin's surface and has been shown in recent meta-analyses to reduce postoperative nausea and vomiting as well as pain and LoS [20^▪^,^21^].

Intestinal surgery impairs intestinal mucosal barrier function and the structure of gut microbiota, which can contribute to impaired gastrointestinal function and infectious complications [22]. Probiotics are beneficial live organisms that contribute to a healthy gut flora and represent an area of increasing interest in perioperative care. Probiotics can be administered alone or in combination with prebiotics, substrates selectively utilized by host microorganisms such as fructo-oligosaccharides, with the combination of pre and probiotics also known as ‘synbiotics’. Meta-analyses in colorectal surgery and cancer patients have shown that the use of probiotics or synbiotics perioperatively not only reduce incidence of ileus but also other postoperative complications such as infectious complications, sepsis, diarrhoea, abdominal collections, diarrhoea, and bloating [23^▪^,^24^]. Overall, there is an emerging consensus that the use of probiotics is well tolerated and beneficial in the postoperative period, reflected by recommendations for their use in recent ERAS guidelines [12^▪▪^].

## NUTRITIONAL REQUIREMENTS AND DIETARY SUPPORT AFTER SURGERY

Even after resumption of gastrointestinal motility, gastrointestinal surgical procedures can have a wide range of effects on gastrointestinal function and lead to complex dietary needs [1]. Indeed, inadequate dietary intake [25] and micronutrient deficiencies [26] can persist for several months following surgery, and such deficiencies are even more marked in those with a complicated postoperative course [27]. Whilst discussion of specific nutritional requirements and adaptations after various procedures is beyond the scope of this review, it is worth noting that in general, in these clinical cohorts, 25–30 kcal/kg/day of energy and of 1–1.5 g/kg/day of protein is recommended by ESPEN [28].

Appropriate implementation of any nutritional intervention necessitates regular assessments of patients’ nutritional status during the perioperative period by a suitably trained member of the healthcare team. The first level of support that can be provided to patients is tailored dietary optimisation and structured dietary counselling. Strategies to optimise in-hospital diet may include meal fortification and encouraging snacks [4,28,29]. Multidisciplinary team input including a dietitian after gastrectomy has been shown to improve outcomes in the acute setting [30] and reduces body weight losses and improves quality of life (QoL) when continued in the postdischarge period [31].

Given the proven benefits of structured dietary counselling and finite availability of specialist dietitian input, there is emerging interest in the use of digital therapeutics in this area. Early feasibility studies of mobile apps supporting abdominal cancer patients with their nutritional needs suggest that mobile apps tracking nutritional intake and nutritional goals are feasible, easy to use and are rated highly for patient satisfaction [32,33]. Determining whether they confer any clinically significant benefit over usual care will require suitably sized, well controlled trials in the future.

## ORAL NUTRITIONAL SUPPLEMENTS

Given the nature of acute postoperative recovery, meeting minimum nutritional requirements with diet alone can be difficult to achieve. It has been shown that even with early permission of oral intake, patients consume inadequate calories and protein in the acute postoperative period; falling substantially short of minimum ESPEN recommendations, with a preference for soft food choices such as soup, jelly and ice cream [4]. Generally, where normal diet is inadequate, but the oral route of feeding is available, oral nutritional supplements (ONS) can be an effective strategy to increase energy and nutrient intake. A number of recent systematic reviews have been published on the efficacy of postoperative ONS in various patient cohorts [34,35^▪^,^36^], including in those with no preoperative malnutrition, suggesting a benefit of routine supplementation [37]. These meta-analyses and recent RCTs [37–39] have consistently demonstrated the benefit of ONS in terms of body weight losses and/or recovery parameters. Interestingly, Rowley *et al.*[34], demonstrated in sub-analysis of an ONS study, that regimens with less than 400 kcal per day were more effective at reducing body weight losses than those providing more than 400 kcal per day, and they attributed this to more marked osmotic effects and consequent diarrhoea with more energy dense preparations. Indeed, ONS therapy is not without side effects such as diarrhoea and appetite loss [38], and outside the context of trials adherence to ONS may be poor [40]. In addition to mitigation of body weight losses, a recent meta-analysis confirmed that ONS initiated after discharge from hospital is associated with an improvement in biochemical parameters representative of health status, specifically serum albumin concentration and haemoglobin [34]. The effect of ONS on other clinically relevant  postoperative outcomes and overall prognosis is more equivocal [35^▪^,^41^^▪▪^]. For example, a recent meta-analysis demonstrated lower postoperative complication rate and shortened LoS with the use of ONS [35^▪^], however, a subsequent large scale multicentre RCT showed that 3 months of postoperative ONS did not result in better compliance with adjuvant therapy and conferred no survival benefit at 3 or 5 years after surgery despite reduced body weight losses [41^▪▪^]. Recent data regarding the effects of ONS on SKM losses is scarce and inconclusive [39,42]. One recent study investigated whether elemental ONS had a beneficial effect on SKM losses but has found no significant benefit [43]. Currently, colorectal ERAS guidelines recommend that ONS should be provided in addition to normal diet routinely for 3–5 days after surgery, aiming to provide 500 kcal and 1.2–1.5 g/kg/day of protein. Patients with identified malnutrition are recommended to continue ONS beyond the point of discharge for at least 10 days [12^▪▪^]. The 1.2–1.5 g/kg/day of protein target recommended by the colorectal ERAS guidelines is in line with ESPEN recommendations. Interestingly, a large-scale international trial in critical care patients has demonstrated that compared to a standard (1.2 g/kg/day) prescription of protein, a high protein prescription of 2.2 g/kg/day (via ONS/EN or parenteral route) did not lead to better outcomes [44]. Additionally, subgroup analysis showed worse outcomes in the high protein group for patients with acute kidney injury and high organ failure scores suggesting that delivering higher doses of protein above current standard practice doses does not provide additional benefit, and in the most unwell patients may even be harmful [44].

## IMMUNONUTRITION

Beyond ONS focussed on energy and macronutrient (i.e., protein) provision, there is a growing body of evidence supporting the perioperative use of immunonutrition. Accepted as nutritional strategies with potential to modulate the activity of the immune system, several meta-analyses support the use of immunonutrition (e.g., preparations containing arginine, n-3 polyunsaturated fatty acids, or glutamine) in gastrointestinal surgery to improve postoperative infectious and all-cause complications [45^▪^,^46^,^47^]. However, these analyses typically include a mixture of both pre and postoperative interventions, with no meta-analysis looking exclusively at postoperative use. Subgroup analysis of a recent meta-analysis concluded that postoperative immunonutrition alone did not elicit improvements in any of the assessed domains, but that combined pre and postoperative immunonutrition reduced incidence of infectious complications and anastomotic leak [46]. A recent comprehensive component network meta-analysis recommends postoperative or combined pre and postoperative administration of immunonutrition [47], and the most recent ERAS and ESPEN guidelines [12^▪▪^,^13^^▪▪^] make a recommendation for their use in the first postoperative week [12^▪▪^].

## ALTERNATIVE ROUTES OF NUTRITIONAL SUPPORT AFTER ABDOMINAL SURGERY

In instances where nutritional needs cannot be met through the oral route, nutritional support in the form of supplementary or total enteral nutrition (EN) or parenteral nutrition (PN) should be considered. Where the enteral route (nasogastric, nasojejunal or jejunostomy) is available, this is preferred over PN due to well established risks associated with PN including line insertion-related, infective and metabolic complications [13^▪▪^]. In some specific cases, there can be a role for routine initiation of postoperative EN. For instance, after oeophagectomy, systematic administration of EN has been shown to be associated with a higher completion rate of adjuvant therapy [48] and is recommended in current ERAS guidelines [18]. More generally, indications for non-oral forms of nutritional therapy should take into consideration the patient's baseline nutritional state, surgical nature and the expected duration of inadequate oral intake. Already malnourished patients should receive EN or PN within 24 h of surgery when oral feeding is not feasible [12^▪▪^]. Otherwise, EN support should be initiated in patients anticipated to be unable to have any oral intake for 5 days, or less than 70% of required intake for a week or more [13^▪▪^]. Standard duration for such nutritional support is not specified in guidelines and depends on ongoing reassessment. Enteral support may need to continue in the postdischarge period, and it has been shown that preoperative malnutrition is associated with a prolonged need for EN after oesophageal cancer resection [49]. Supporting the potential benefit of specific forms of EN, a systematic review in postgastrectomy patients demonstrated an additional benefit of enteral immunonutrition versus standard enteral nutrition with regards to infectious complication rates [50].

Recent meta-analysis comparing exclusive EN versus PN after esophagectomy has demonstrated a smaller decrease in albumin levels 1 week postoperatively with PN, but also significantly higher total complication rate and longer postoperative LoS [51^▪^]. However, other recent meta-analyses advocate for the benefits of early supplementary PN arguing for the potential of a combined approach to facilitate rapid delivery of nutrients whilst reducing gastrointestinal burden and maintaining beneficial gastrointestinal flora and mucosal integrity [52,53^▪^]. For instance, a recent meta-analysis of RCTs in patients post-gastrointestinal surgery has demonstrated that a combined approach of EN and PN therapy led to shorter LoS and fewer abdominal symptoms than EN alone [53^▪^]. However, given the well established risks and higher cost associated with PN, these results should be interpreted with caution. Routine PN is not recommended in current clinical guidelines, but supplementary PN is recommended if the oral/enteral nutrition routes fail to provide at least 50% of energy requirements after 3–4 days [13^▪▪^].

## EXERCISE INTERVENTIONS AFTER ABDOMINAL SURGERY

In addition to nutrition, mobility and exercise-based interventions are another main pillar of rehabilitation efforts. Although clinical rehabilitation studies frequently explore exercise and nutritional interventions in isolation, there is data supporting the synergistic relationship between nutritional and contractile interventions in clinical settings [54]. For abdominal surgery patients, the mainstay of inpatient exercise interventions in current practice is limited to early mobilisation, ideally on the day of surgery and for at least 3 h per day after postoperative day 1 [12^▪▪^]. Whilst early mobilisation has been shown to be associated with earlier recovery of gastrointestinal function, it does not necessarily lead to higher overall inpatient step count and confers no benefit in terms of morbidity or LoS [55]. Observational studies of inpatient populations have demonstrated that baseline malnutrition and reduced inpatient step count are associated with an increased risk of SKM losses [56] and unplanned readmission [57]. However, even relatively modest step/physical activity targets are difficult to achieve in the immediate postoperative period and reported inpatient step counts after gastrointestinal surgery are typically less than 1000/day during the first week after surgery [5,58]. Implementation of structured exercise programmes in the acute postoperative, inpatient period is challenging due to (e.g.,) pain, nausea and indwelling lines. Nevertheless, an RCT in colorectal patients showed that early structured, progressive resistance exercise resulted in reduced LoS, higher perceived readiness for discharge and SKM preservation [59]. An emerging area of interest for inpatient exercise mimetic therapy after surgery is the use of neuromuscular electrical stimulation (NMES). Postoperative quadriceps NMES in the immediate 4 days after colorectal surgery [58] and for 4 weeks after liver transplant [60] has been shown to reduce SKM losses. Further, a systematic review of postoperative quadriceps NMES concluded that it results in increases in lower limb strength after cardiac surgery, yet further evidence is necessary in abdominal surgery [61^▪^]. Additionally, there are currently no studies looking at the combined impact of postoperative NMES and nutritional support.

Following discharge from hospital a wider variety of exercise options become available. A meta-analysis looking at postoperative exercise programmes in colorectal, breast and prostate cancer patients concluded an improvement in cardiorespiratory function, functional capacity and QoL, but noted a lack of high-quality, large trials [62]. In addition to the varied surgical cohorts, the exercise programmes considered in this analysis were also heterogenous including aerobic, resistance or combination training; somewhat limiting the ability for recommendations to be drawn from this work. A more specific meta-analysis of postoperative aerobic exercise training after abdominal cancer resections demonstrated a significant improvement in physical performance (6-min walk distance), albeit with a variable effect on QoL [63]. In terms of SKM preservation, a meta-analysis looking at exercise programmes postoesophagectomy demonstrated no benefit [64^▪^]. Whilst the effects of combined nutritional and exercise interventions are of interest and clearly relevant given the established interaction between protein nutrition and contractile activity for muscle mass maintenance, such trials in abdominal surgery patient cohorts are scarce. A prospective study utilising a 1-month exercise programme plus oral branched-chain amino acid supplements in elderly patients postgastrectomy demonstrated improvements in skeletal mass index and QoL scores [65]. However, no control group was included and as such firm conclusions are difficult to reach.

## CONCLUSION

**FIGURE 1:**
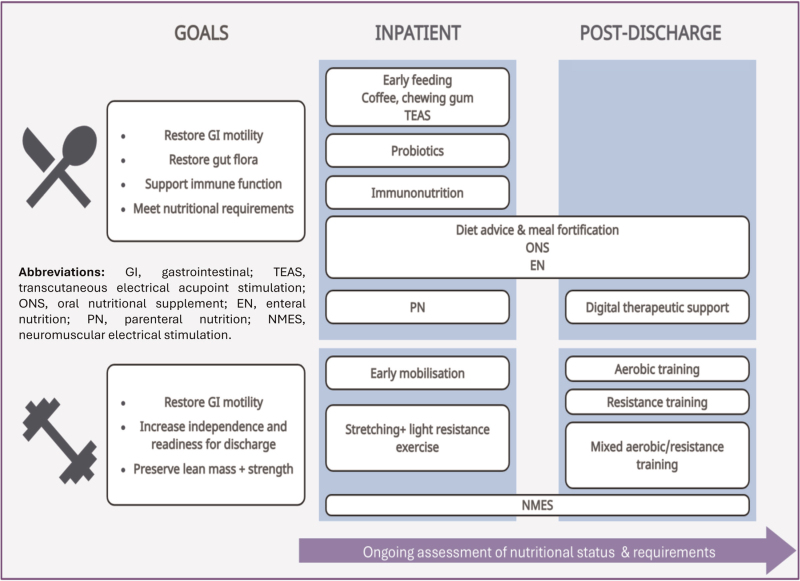
Schematic diagram illustrating the rehabilitation modalities discussed in the review. The upper panels illustrate goals of nutritional interventions and strategies available in the inpatient and post-discharge period. The lower panels illustrate goals of exercise interventions and strategies available in the inpatient and post-discharge period. Abbreviations: GI, gastrointestinal; TEAS, transcutaneous electrical acupoint stimulation; ONS, oral nutritional supplement; EN, enteral nutrition; PN, parenteral nutrition; NMES, neuromuscular electrical stimulation

Primary aims of early postoperative rehabilitation include return of gastrointestinal function and meeting nutritional requirements to improve postoperative outcomes. This requires ongoing nutritional assessment and early intervention where indicated. A further consideration includes supporting immune function, with growing emphasis on the use of immunonutrition. Following discharge, ongoing nutritional supplementation can be of benefit for select patients, with evidence to support the beneficial impact of continued dietetic input. Options for exercise-based interventions in early recovery are limited including with adjuvant nutritional support; NMES is an area of emerging interest in this regard. Postdischarge exercise programmes have been shown to improve functional outcomes and QoL, but further evidence on the effectiveness of multimodal (i.e., exercise-plus-nutrition) interventions, specifically after abdominal surgery, is still required. Another area of interest for further research is the use of personalised mobile nutrition apps. Lastly, much of the existing literature around nutritional rehabilitation focuses on body weight loss mitigation as the outcome of interest; assessment of benefits beyond body weight maintenance such as clinical prognosis, functional outcomes and SKM mass gains are needed (Fig. 1).

## Acknowledgements

*This manuscript has been seen, reviewed and approved by all contributing authors. There are no other acknowledgements for this work.*

### Financial support and sponsorship

*This work was supported by the Medical Research Council (MR/P021220/1 and MR/X005240/1).*

### Conflicts of interest

*No author has any conflict of interest to declare.*

## REFERENCES AND RECOMMENDED READING

Papers of particular interest, published within the annual period of review, have been highlighted as:

▪ of special interest
▪▪ of outstanding interest
